# Reply to Battke et al. Comment on “Park et al. Lessons Learned from Translating Genome Sequencing to Clinical Routine: Understanding the Accuracy of a Diagnostic Pipeline. *Genes* 2024, *15*, 136”

**DOI:** 10.3390/genes15101323

**Published:** 2024-10-14

**Authors:** Joohyun Park, Marc Sturm, Tobias B. Haack

**Affiliations:** 1 Institute of Medical Genetics and Applied Genomics, University of Tübingen, 72076 Tübingen, Germany; joohyun.park@med.uni-tuebingen.de; 2 Center for Rare Diseases, University of Tübingen, 72076 Tübingen, Germany

We are writing in response to the comment [1] by Battke et al. on our paper “Lessons learned from translating genome sequencing to clinical routine: understanding the accuracy of a diagnostic pipeline” [2]. Concerns were raised regarding the relevance of our study for understanding the benefits of genome sequencing (GS).

One of the main goals of our paper was to share our quality control schemes and technical validating strategies, highlighting their importance when implementing GS as primary diagnostic method and when comparing exome sequencing (ES) to GS. For a uniform data presentation from the technical point of view, we selected all technical and diagnostic information from samples sequenced in 2022, which were processed using the same platforms and sample preparation techniques. In this context, we reported coverage uniformity and variant calling benchmarks where GS showed superior performance compared to our ES, even in the coding regions. Battke et al. criticized that the cohorts are too divergent in size and age to draw any conclusions, and the use standard exome enrichment kits. Indeed, the phenotypes, age groups, and the use of trio-analysis varied significantly between the ES and GS cohorts, and not all samples underwent both ES and GS, making direct comparison challenging. However, we have openly discussed these limitations in our paper. Despite these constraints, we believe readers may still find value in observing the true diagnostic outcomes of both separate cohorts. Given that non-coding variants and certain structural variations (SVs) are usually not detectable in standard exome kits, we suggested that approximately 9% of solved cases in GS were such variations and potential bottlenecks of standard ES. We double-checked the non-coding variants, and we are sure that they are not reliably detectable in exome sequencing. Battke et al. also recommended expressing the absolute increase in diagnostic yield as 1.8% instead of the relative increase of 9%. We agree that although our presentation of the increased yield is correct, the absolute gain should be added to avoid misunderstandings. Newer or custom-designed enrichment kits could address some known shortcomings of ES, covering clinically relevant intronic regions, which is surely worth mentioning. Meanwhile, we have also established a Twist-based custom exome kit to improve target coverage. However, in our experience, samples processed with the custom exome kit still have up to four times more low coverage bases (<20×) samples in the coding/splicing regions of OMIM genes than genome samples. Our center previously published GS diagnostic yield data of 1000 patients with inherited eye diseases, estimating potential changes that might have been missed between 1.7% to 8.5% depending on the used exome kit [3]. In this disease group, SVs and non-coding variants were observed in 12.7% of solved diagnoses. Depending on the exome or panel enrichment, 5–10% additional diagnoses were estimated when using GS. A similar estimation was made by recent observation by Wojcik et al., in which 8.2% of families required GS to identify causal variants [4]. Despite the disparate cohort sizes and ages, our estimation seems to be similar to those of other GS studies.

We would like to take this opportunity to highlight another major benefit of GS that should not be missed, which is its full availability for reanalysis. The importance of non-coding variations, e.g., in introns, regulatory elements, or long non-coding RNAs, is increasingly recognized, and our knowledge of these variants is constantly growing. Additionally, in cases with a specific suspected diagnosis, particularly in the presence of a heterozygous pathogenic variant in an autosomal recessive disease, investigating non-coding variants can be essential. Such variants may be clarified through further investigations (e.g., functional evaluation, segregation studies, and transcriptome analysis). Thus, GS also offers the possibility of detecting both recently described and new pathogenic non-coding variants, which might represent major causes, e.g., neurodevelopmental disorders, as suggested by recent publications on RNU4-2 [5,6].

We are pleased to see that GS is increasingly used for various purposes, and with the growing availability of bioinformatic analysis tools, clinical evaluation has become easier. However, implementing GS in clinical diagnostics can be challenging, as it requires experts from different fields who need to familiarize themselves with the technology. Before a laboratory transitions from ES to GS, various factors, such as cost, infrastructure, and staffing, need to be considered. Numerous studies, including those cited in our paper, have demonstrated the additional benefits of GS. We are confident that our study contributes constructively to this body of knowledge. With GS, more variant types can be detected and even more precisely than with ES, and that which cannot currently be identified with short-read GS will likely be detectable by long-read GS.
